# Null Broadening Robust Adaptive Beamforming Algorithm Based on Power Estimation

**DOI:** 10.3390/s22186984

**Published:** 2022-09-15

**Authors:** Zhenhua Yu, Weijia Cui, Yuxi Du, Bin Ba, Mengjiao Quan

**Affiliations:** 1 School of Cyber Science and Engineering, Zhengzhou University, Zhengzhou 450002, China; 2 National Digital Switching System Engineering & Technological Research Center, Zhengzhou 450001, China

**Keywords:** robust adaptive beamforming, null broadening, power estimation, virtual interference

## Abstract

In order to solve the problem of severely decreased performance under the situation of rapid moving sources and unstable array platforms, a null broadening robust adaptive beamforming algorithm based on power estimation is proposed in this paper. First of all, we estimate the interference signal power according to the characteristic subspace theory. Then, the correspondence between the signal power and steering vector (SV) is obtained based on the orthogonal property, and the interference covariance matrix (ICM) is reconstructed. Finally, with the aim of setting virtual interference sources, null broadening can be carried out. The proposed algorithm results in a deeper null, lower side lobes and higher tolerance of the desired signal steering vector mismatch under the condition of low complexity. The simulation results show that the algorithm also has stronger robustness.

## 1. Introduction

Adaptive beamforming is a classic problem in array signal processing, which has been widely used in radar, sonar, communication, microphone array processing, medical imaging, and other fields [1]. Adaptive beamforming can effectively inhibit interference and noise from other directions while receiving the desired signal [2,3]. When the desired signal is accurately known, beamforming has an excellent ability to suppress interference. However, because there is signal SV mismatch in actual situations, the performance degradation of adaptive beamforming will occur [4]. At the same time, the desired signal (DS) component usually exists in training data, and a slight mismatch in the high interference-plus-noise ratio (SINR) also results in a serious decrease in the adaptive beamforming performance. In recent years, a series of robust adaptive beamforming (RAB) algorithms for steering vector mismatch and covariance matrix estimation inaccuracy have been proposed, such as the diagonal loading (DL) algorithm [5], the eigenspace projection (EIG) algorithm [6], the robust adaptive beamforming algorithm based on SV uncertainty set [7], and the interference-plus-noise covariance matrix (INCM) reconstruction method [8,9,10]. However, the nulls generated by the above adaptive beamformers are all very narrow. When the interference direction cannot be accurately obtained due to the fast movement of the interference source or the vibration of the antenna platform, the actual interference may no longer be null, which will cause the obtained weight vector to be unable to suppress actual interference.

To solve the above problems, a large number of null broadening methods have been proposed. Among them, the Covariance matrix taper (CMT) is a classic algorithm, which essentially uses a matrix related to null width to enhance the coordinating differential matrix. It was first proposed by Milloux [11], and it assumes that there is a discrete, equivalent interval, and equivalent power virtual interference in the vicinity of the actual interference source to expand the null. Zatman [12] assumes that the actual interference source has a certain bandwidth to expand the null. Guerci [13] introduced a unified concept of covariance matrix taper, which proves that the CMT algorithm is a solution of the least variance optimal beamforming. This type of algorithm has low computational complexity, but with the increase in the amount of virtual interference, the depth of the null will gradually decrease, and the effect of interference suppression will be weakened. In recent years, some improved algorithms of CMT have been proposed. In [14], the authors proposed an algorithm for combining variable diagonal loading and the CMT algorithm. This algorithm has higher robustness, but its performance gradually decreases with the improvement in the input signal-to-noise ratio (SNR). Liu et al. [15] proposed a method of flexible null broadening that improves the degree of freedom of the algorithm. In [16], the authors combined the CMT algorithm with the amplitude constraint, and deepened the null at the same time as the null broadening. However, the robustness of SV mismatch is low in high SNR. A method based on projection transformation and diagonal loading was proposed in [17], which has a deeper null. Yang et al. [18] combined the PDNBB algorithm with the uncertain set optimization algorithm, which improved the robustness of SV mismatch.

However, the above algorithms do not completely remove the DS component. The reconstructed class algorithm proposed in recent years effectively removed the impact of the DS component by setting the angular interval in a space. In [19], the authors proposed a null broadening beamforming algorithm for covariance matrix reconstruction and similarity constraints (CMRSC). This algorithm effectively eliminated the DS component by reconstructed thought, and the effect was still close to the theoretical value during high-input SNR. On the basis of the CMRSC algorithm, ref. [20] introduced power parameters to regulate the depth of the null, and the degree of interference suppression was subsequently stronger. However, the computational complexities of the above-mentioned reconstruction algorithms are high, and the applications are limited in an environment with a high degree of real-time requirements. In [21], the authors decreased algorithm complexity with the reconstruction of INCM by using the simplified power spectral density function. The null broadening method of the derivative constraint was proposed in [22,23], but its computational complexity was high. In [24], the quadratic constraint of [25] was substituted by a set of linear constraints, thereby causing null broadening in the interference direction. In [26], the authors proposed a null broadening beamforming algorithm based on decomposition and iterations. The algorithm had stronger robustnesss against the SV mismatch and nonstationary interference. Zhang et al. [27] proposed a robust beamforming algorithm for anti-fast-moving interference based on minimal dispersion (MD) criteria, which was better able to receive non-Gaussian signals.

A new null broadening algorithm is proposed by combining the power estimation and the idea of virtual interference sources. We derive the relationship between feature vectors and direction vectors through feature space theory, and obtain the approximate relationship between signal power and the eigenvalue. Furthermore, then, we reconstruct the ICM. At the same time, the introduction of the adjustment factor increases the null depth and results in the better suppression of interference. The simulation results show that the algorithm proposed in this paper is more robust to large SV mismatch while deepening the null.

The main contributions of this paper are summarized as follows:

(a) A new null broadening method is proposed based on subspace theory, which has a deeper null and a lower side lobe level.
(b) An estimation of interference signal power is proposed through the relationship between the signal direction vector and the eigenvalue and feature vector, which greatly reduces the complexity of the algorithm.
(c) We give the performance comparisons of the proposed and relevant beamformers using typical experiments. Simulation results show that the proposed algorithm has good performance both under ideal conditions and with DOA errors.

Other parts of this paper are organized as follows: Section 2 describes the signal model. Section 3 estimates interference power and null broadening. Section 4 gives an algorithm simulation experiment under different conditions. Section 5 summarizes the work of this paper.

## 2. The Signal Model

Consider a uniform linear array (ULA) with *M* omnidirectional receiving sensors. Assume that there are *K* far-field narrowband source-independent signals from different central directions $\theta =\left(\theta_{1},\theta_{2},\cdots ,\theta_{K}\right)$ to impinge on the array. The array spacing *d* is set as half of the wavelength of the incident signal (d=λ/2). The desired signal is irradiated from the direction $\theta_{1}$ to the array, the remaining K−1 interference signals come from the direction $\theta_{2},\cdots ,\theta_{K}$, and then the array stream is represented as

(1) $$A=\left[a\left(\theta_{1}\right),\cdots ,a\left(\theta_{K}\right)\right]$$

where $a(\theta_{i})$ is a directed vector and can be written as

(2) $$a\left(\theta_{i}\right)={\left(1,e^{-j2\pi \frac{d_{1}\sin\theta_{i}}{\lambda }},\cdots ,e^{-j2\pi \frac{d_{M-1}\sin\theta_{i}}{\lambda }}\right)}^{T}$$

where $d_{m}$ represents the distance between the m+1th antenna in the antenna array relative to the first antenna, and $d_{m}\in P$, P is the location collection of physical chains.

The received signal vector x(t) at the time of *t* can be modeled as

(3) $$x\left(t\right)=a\left(\theta_{1}\right)s_{1}\left(t\right)+\sum_{i=2}^{K}a\left(\theta_{i}\right)s_{i}\left(t\right)+n\left(t\right)$$

where $a(\theta_{i}),i=1,\cdots ,K$ and $s_{i}\left(t\right),i=1,\cdots ,K$ are the signal steering vectors and waveforms, and n(t) is additive White Gaussian noise, which is independent of any signal, with zero mean and fixed variance ${\sigma_{n}}^{2}$. Theoretically, the covariance matrix of the array is

(4) $$\begin{array}{cc} R & =E\left\{x\left(t\right)x^{H}\left(t\right)\right\}=R_{s}+R_{i}+R_{n}\\ & =\sigma_{1}^{2}a\left(\theta_{1}\right)a^{H}\left(\theta_{1}\right)+\sum_{i=2}^{K}\sigma_{i}^{2}a\left(\theta_{i}\right)a^{H}\left(\theta_{i}\right)+\sigma_{n}^{2}I_{M} \end{array}$$

where ${\left(\cdot \right)}^{H}$ is a conjugate transposition, E(⋅) is the expected value, $\sigma_{1}^{2}$ is the desired signal power, $\sigma_{i}^{2}$ is the power of the interference signal, $\sigma_{n}^{2}$ is the power of the noise signal, and $I_{M}$ is the identity matrix.

The INCM is expressed as

(5) $$\begin{array}{c} R_{i+n}=\sum_{i=2}^{K}\sigma_{i}^{2}a\left(\theta_{i}\right)a^{H}\left(\theta_{i}\right)+\sigma_{n}^{2}I_{M} \end{array}$$

With MVDR guidelines, we can achieve the best beamforming by addressing the following issues:

(6) $$\begin{array}{c} \left\{\begin{array}{c} \min_{w}\;w^{H}R_{i+n}w\\ s.t.\;w^{H}a\left(\theta_{1}\right)=1 \end{array}\right. \end{array}$$

Therefore, the optimal weight vector can be expressed as

(7) $$\begin{array}{c} w=\frac{R_{i+n}^{-1}a\left(\theta_{1}\right)}{a^{H}\left(\theta_{1}\right)R_{i+n}^{-1}a\left(\theta_{1}\right)} \end{array}$$

In practical applications, the INCM $R_{i+n}$ is difficult to obtain. $R_{i+n}$ is often replaced with the sample covariance matrix (SCM) as

(8) $$\begin{array}{c} {\overset{\Lambda }{R}\;}_{x}=\frac{1}{Q}\sum_{t=1}^{Q}x\left(t\right)x^{H}\left(t\right) \end{array}$$

where Q is the total number of snapshots.

We use ${\overset{\Lambda }{R}\;}_{x}$ instead of $R_{i+n}$, which causes the algorithm reception signals to contain a desired signal that results in a decrease in algorithm performance. With the improvement in SNR, the proportion of the desired signal component gradually increases, resulting in a serious decrease in beamforming performance.

*Q* snapshots beamforming output as

(9) $$\begin{array}{c} y\left(t\right)=w^{H}x\left(t\right),\left(t=1,2,\cdots ,Q\right) \end{array}$$

We usually use output SINR to judge the performance of beamforming, which can be expressed as

(10) $$\begin{array}{c} \mathrm{SIN}R≜\frac{\sigma_{1}^{2}|w^{H}a\left(\theta_{1}\right){|}^{2}}{w^{H}R_{i+n}w} \end{array}$$

## 3. The Proposed Algorithm

In this section, a novel null broadening RAB algorithm based on signal power estimation is proposed. We estimate the relationship between the signal power and eigenvalues through the subspace method, and then set the null broadening by setting up the source of virtual interference.

### 3.1. Doa Estimation

Assuming that the desired signal direction vector is known, the interference direction is estimated by the DOA. We use the MUSIC algorithm to calculate the direction of interference.

The theoretical covariance matrix of received signal x(t) can be written as:

(11) $$\begin{array}{c} R=\sum_{i=1}^{K}\sigma_{i}^{2}a_{i}a_{i}^{H}+\sigma_{n}^{2}I_{M} \end{array}$$

Because the spatial source signals are irrelevant, R is a nonsingular matrix. We have $R^{H}=R$. Therefore, the characteristic decomposition of R can be written as

(12) $$\begin{array}{cc} R=U\Sigma U^{H} & =\sum_{i=1}^{K}\lambda_{i}e_{i}e_{i}^{H}+\sum_{j=K+1}^{M}\lambda_{j}e_{j}e_{j}^{H}\\ & =U_{S}\Sigma_{S}U_{S}^{H}+U_{N}\Sigma_{N}U_{N}^{H} \end{array}$$

where λ are the eigenvalues of R in descending order from large to small, as follows:

(13) $$\lambda_{1}\geq \lambda_{2}\geq \cdots >\lambda_{k}>\lambda_{k+1}=\cdots =\lambda_{M}=\sigma_{n}^{2}$$

The first *K* eigenvalues are related to the signals, and the values are greater than $\sigma_{n}^{2}$. Their corresponding eigenvectors are $e_{1},e_{2},\cdots ,e_{K}$, and they constitute the signal subspace $U_{S}\in C^{M\;\;\;\times \;\;\;K}$. The last M−K small eigenvalues are completely dependent on the noise, and the values are equal to $\sigma_{n}^{2}$. The corresponding feature vector constitutes the noise subspace $U_{N}\in C^{M\;\;\;\times \;\;\;(M-K)}$, $\Sigma_{S}=diag\left(\lambda_{1},\cdots ,\lambda_{K}\right)$, $\Sigma_{N}=diag\left(\lambda_{K+1},\cdots ,\lambda_{M}\right)$. Hereinafter, we give some properties about the feature subspace under independent signal conditions, in preparation for subsequent algorithms and their theoretical analysis: $U_{N}^{H}a\left(\theta_{i}\right)=0,i=1,2,\cdots ,K$, $e_{i}^{H}e_{i}=1$, $a_{i}^{H}a_{i}=M$, $U^{H}U=UU^{H}=I_{M}$.

We can obtain the array spatial spectrum function through the orthogonal relationship between the noise feature vector and the signal vector as

(14) $$\begin{array}{c} P_{MUSIC}\left(\theta \right)=\frac{1}{a^{H}\left(\theta \right)U_{N}U_{N}^{H}a\left(\theta \right)} \end{array}$$

By changing θ, the arrival angle of the signals is estimated by looking for peaks.

### 3.2. Signal Power Estimation

As shown in Formula (12), $\Sigma_{N}=\sigma_{n}^{2}I_{\left(M-K\right)}$. Therefore, we have

(15) $$\begin{array}{cc} \; & R=U_{S}\Sigma_{S}U_{S}^{H}+U_{N}\Sigma_{N}U_{N}^{H}\\ \; & \;=U_{S}\Sigma_{S}U_{S}^{H}-U_{S}{\hat{\Sigma }}_{N}U_{S}^{H}+U_{S}{\hat{\Sigma }}_{N}U_{S}^{H}+U_{N}\Sigma_{N}U_{N}^{H}\\ \; & \;=U_{S}\left(\Sigma_{S}-{\hat{\Sigma }}_{N}\right)U_{S}^{H}+\sum_{i=1}^{K}\sigma_{n}^{2}e_{i}e_{i}^{H}+\sum_{j=K+1}^{M}\sigma_{n}^{2}e_{j}e_{j}^{H}\\ \; & \;=U_{S}\left(\Sigma_{S}-{\hat{\Sigma }}_{N}\right)U_{S}^{H}+\sigma_{n}^{2}\sum_{q=1}^{M}e_{q}e_{q}^{H}\\ \; & \;=U_{S}\left(\Sigma_{S}-{\hat{\Sigma }}_{N}\right)U_{S}^{H}+\sigma_{n}^{2}UU^{H}\\ \; & \;=U_{S}\left(\Sigma_{S}-{\hat{\Sigma }}_{N}\right)U_{S}^{H}+\sigma_{n}^{2}I_{M} \end{array}$$

${\hat{\Sigma }}_{N}=\sigma_{n}^{2}I_{K}$; therefore, contrasting Formulas (11) and (15), we can obtain

(16) $$\begin{array}{c} \sum_{i=1}^{K}\sigma_{i}^{2}a_{i}a_{i}^{H}=U_{S}\left(\Sigma_{S}-\hat{\Sigma_{N}}\right)U_{S}^{H}=\sum_{i=1}^{K}(\lambda_{i}-\sigma_{n}^{2})e_{i}e_{i}^{H} \end{array}$$

We know from the properties of the feature subspace that

(17) $$\begin{array}{c} span\left\{e_{1},e_{2},\cdots ,e_{K}\right\}=span\left\{a_{1},a_{2},\cdots ,a_{K}\right\} \end{array}$$

where $span\{e_{1},e_{2},\cdots ,e_{K}\}$ is a signal subspace formed by the eigenvectors corresponding to the big eigenvalue of the covariance matrix. $span\{a_{1},a_{2},\cdots ,a_{K}\}$ is a subspace of the SVs expansion of the incident signal. The feature vector $e_{i}\left(i=1,\cdots ,K\right)$ is linear independent. Hence, we have

(18) $$\begin{array}{c} a_{i}=l_{i1}e_{1}+l_{i2}e_{2}+\cdots +l_{iK}e_{K} \end{array}$$

Because each direction vector $a_{i}$ corresponds to a unique signal, and each large eigenvalue corresponds to a unique signal, by solving

(19) $$\begin{array}{c} \max\{cor\left(a_{i},e_{s}\right)=\frac{\left|vec{\left(a_{i}\right)}^{H}vec\left(e_{s}\right)\right|}{∥vec(a_{i})∥\;∥vec(e_{s})∥}\},s=1,2,\cdots ,K \end{array}$$

the eigenvector $e_{s}$ with the highest correlation with $a_{i}$ can be obtained. Therefore, $a_{i}$ and $e_{s}$ approximately meet the formula

(20) $$\begin{array}{c} \left\{\begin{array}{c} a_{i}\approx l_{is}e_{s}\\ a_{i}^{H}\approx l_{is}^{*}e_{s}^{H} \end{array}\right.,s\in \left\{1,2,\cdots ,K\right\} \end{array}$$

Furthermore, there is a unique correspondence between *i* and *s*. Through the above analysis, we have the following formula

(21) $$\begin{array}{c} M=a_{i}^{H}a_{i}\approx l_{is}^{*}l_{is}e_{s}^{H}e_{s}=l_{is}^{*}l_{is},i,s\in \left\{1,2,\cdots ,K\right\} \end{array}$$

Combining Formulas (16) and (20), we can obtain

(22) $$\begin{array}{c} \sum_{i=1}^{K}(\lambda_{i}-\sigma_{n}^{2})e_{i}e_{i}^{H}\approx \sum_{i=1}^{K}\sigma_{i}^{2}l_{is}l_{is}^{*}e_{s}e_{s}^{H} \end{array}$$

The left side of the Formula (22) is multiplied to $e_{s}^{H}$, and the right is multiplied to $e_{s}$. We can obtain

(23) $$\begin{array}{cc} e_{s}^{H}\sum_{i=1}^{K}(\lambda_{i}-\sigma_{n}^{2})e_{i}e_{i}^{H}e_{s} & \approx e_{s}^{H}\sum_{i=1}^{K}\sigma_{i}^{2}l_{is}l_{is}^{*}e_{s}e_{s}^{H}e_{s}\\ \;e_{s}^{H}\left(\lambda_{s}-\sigma_{n}^{2}\right)e_{s}e_{s}^{H}e_{s} & \approx e_{s}^{H}\sigma_{i}^{2}l_{is}l_{is}^{*}e_{s}e_{s}^{H}e_{s}\\ \;(\lambda_{s}-\sigma_{n}^{2}) & \approx \sigma_{i}^{2}l_{is}l_{is}^{*}\\ \;\frac{\left(\lambda_{s}-\sigma_{n}^{2}\right)}{M} & \approx \sigma_{i}^{2},\;i,s\in \left\{1,2,\cdots ,K\right\} \end{array}$$

We can find the corresponding relationship between direction vector $a_{i}$ and interference power $\sigma_{i}^{2}$ according to Formula (20). Since the expected signal SV is accurately known, the reconstructed interference covariance matrix (ICM) can be obtained as

(24) $$\begin{array}{c} {\tilde{R}\;}_{i}=\sum_{i=2}^{K}\frac{\left(\lambda_{s}-\sigma_{n}^{2}\right)}{M}a_{i}a_{i}^{H}\;i,s\in \left\{2,3,\cdots ,K\right\} \end{array}$$

The effectiveness of the proposed ICM reconstruction algorithm can be measured by the correlation coefficient between $R_{i}$ and $\tilde{R}{\;}_{i}$, which is defined as

(25) $$\begin{array}{c} cor\left(R_{i},\tilde{R}\;{}_{i}\right)=\frac{\left|vec{\left(R_{i}\right)}^{H}vec\left(\tilde{R}\;{}_{i}\right)\right|}{∥vec(\overset{}{R_{i}}\;)∥\;∥vec(\tilde{R}\;{}_{i})∥} \end{array}$$

The operator $vec\left(\cdot \right)$ stands for the vectorization of a matrix.

**Figure 1:**
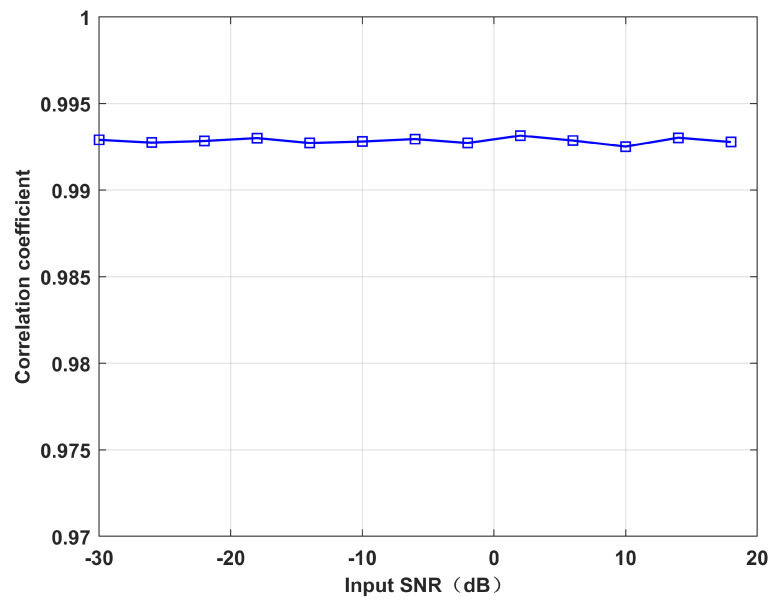
Correlation coefficient versus input SNR.

Figure 1 illustrates the correlation coefficient between the reconstructed ICM and the true ICM under different input SNR. Setting the input SNR as [−30,20], other simulation conditions are the same as simulation 5 in Section 4. The correlation coefficient of the proposed algorithm is approximately 0.993, which means that our algorithm is very efficient.

### 3.3. Null Broadening

Since the DS $\theta_{1}$ is known, we set *L* virtual interference sources with equal intervals and the same power near each actual interference source $\theta_{2},\theta_{3},\cdots ,\theta_{K}$. Let us set *L* to be even for simplicity (which does not affect the results), and set the interference interval as φ. At this time, the null width at each interference is φL. The direction of the actual interference source and the virtual interference source are

(26) $$\begin{array}{cc} \hat{\theta_{i}}=\{\theta_{i}-\frac{\varphi L}{2},\; & \theta_{i}-\frac{\varphi (L+2)}{2},\cdots ,\theta_{i},\cdots ,\theta_{i}+\frac{\varphi L}{2}\}\;,\;\\ i & =2,3,\cdots ,K \end{array}$$

At the same time, the power parameter *k* is introduced to improve the null depth by reducing the power ratio of the noise covariance matrix in INCM. Due to the existence of the actual error, we take the mean of small eigenvalues as noise power, which is

(27) $$\begin{array}{c} \hat{\sigma_{n}^{2}}=\frac{1}{M-K}\sum_{j=K+1}^{M}\lambda_{j} \end{array}$$

Thus, we reconstruct the new INCM to

(28) $$\begin{array}{c} {\hat{R}}_{i+n}=\sum_{i=1}^{K}\sigma_{i}^{2}a\left(\hat{\theta_{i}}\right)a^{H}\left(\hat{\theta_{i}}\right)+k\hat{\sigma_{n}^{2}}I_{M} \end{array}$$

The reconstructed INCM ${\hat{R}}_{i+n}$ is substituted into the Formula (7) and the weight vector of the beamforming in this paper is

(29) $$\begin{array}{c} w=\frac{{\hat{R}}_{i+n}a\left(\theta_{1}\right)}{a^{H}\left(\theta_{1}\right){\hat{R}}_{i+n}^{-1}a\left(\theta_{1}\right)} \end{array}$$

**Figure 2:**
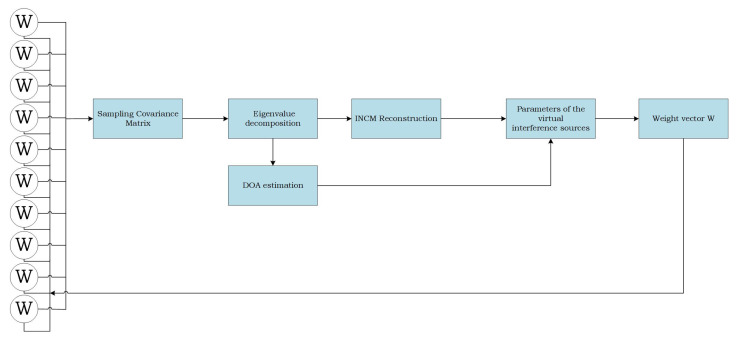
Flow of the proposed beamformer algorithm.

The implementation of the proposed algorithm is summarized in Table 1. The algorithm flow is shown in Figure 2.

The complexity of the algorithm in this article is mainly derived from the eigenvalue decomposition, null broadening and weight vector. The computational complexity of the eigenvalue decomposition is $O(M^{3})$, and the complexity of the interference estimate is $O(JM^{2})$, where *J* is the number of virtual interference sources. The complexity of calculating the weight is $O(\left(M^{2}+2M+1\right)M)$. Therefore, the complexity of this algorithm is $O(\max\left(M^{3},JM^{2},\left(M^{2}+2M+1\right)M\right))$.

The complexity of the CMRSC algorithm is $O(\max\left(2M^{2},PM^{2},\left(M^{2}+2M+1\right)M\right))$, where *P* is the number of discrete sampling points in the approximate integral step, and P≫J,P≫M. The algorithm used in the literature [20] is $O(\max\left(M^{3},PM^{2},\left(M^{2}+2M+1\right)M\right))$. The complexity of the PDL algorithm is $O(\max\left(M^{3},6M^{2},\left(M^{2}+2M+1\right)M\right))$, and the CMT algorithm is $O(\max\left(M^{3},M^{2},\left(M^{2}+2M+1\right)M\right))$. Therefore, the PDL algorithm and the CMT algorithm belong to the same arithmetic level, far below the complexity of the CMRSC algorithm and the literature [20] algorithm. Table 2 intuitively presents the complexity comparisons of each algorithm.

## 4. Simulation Results

We simulate the uniform linear array with the element number of M=10, d=λ/2. The additive white Gaussian noise is modified to be independent noise with unit variance and a value of zero. Simulations determine the number of snapshots to be 100, in addition to verifying the effect of the number of snapshots on the performance of the algorithm. The input signal incidents from the direction of $\theta_{1}=20{}^{\circ }$. All simulations other than the simulation of the input signal-to-noise ratio are set to input SNR=10 dB. Input INR are 30 dB from $\theta_{2}=-10{}^{\circ }$ and $\theta_{3}=50{}^{\circ }$. To ensure the accuracy of simulation, we conducted 200 Monte Carlo experiments to avoid error disturbance.

### 4.1. Effect of Different Parameters of this Paper Algorithm

Simulations 1, 2, and 3 perform control variable simulation comparisons for the power parameter *k*, virtual interference interval φ and null width of this paper, and verify the influence of each parameter of the algorithm in terms of the algorithm performance, respectively.

**Simulation** **1.** *Performance with a variable power parameter, k.*

**Figure 3:**
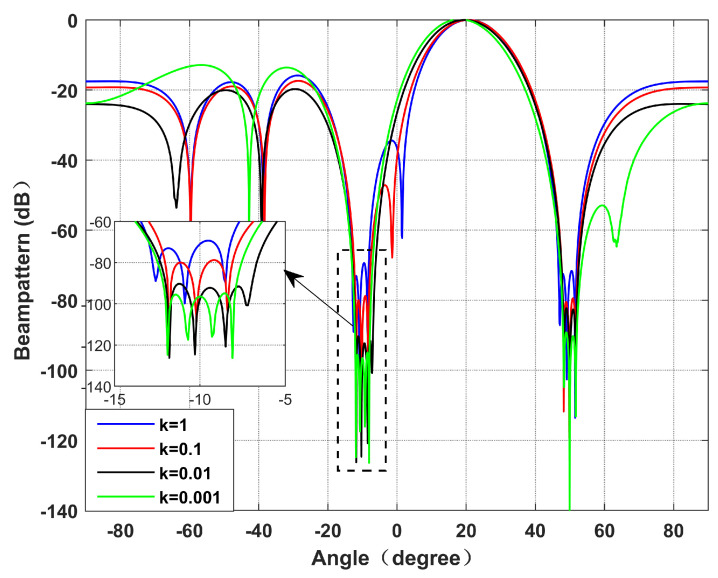
Beamforming diagram with different values of *k*. Simulation 1.

**Figure 4:**
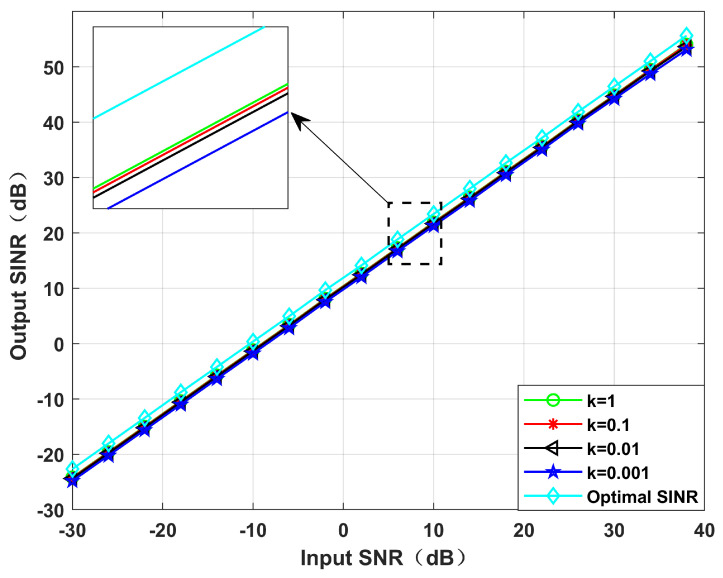
Output SINR versus the input SNR for different values of *k*. Simulation 1.

Figure 3 and Figure 4 simulate the beamforming diagram and the output SINR changes with the input SNR during differences in the power parameter *k*. We set the virtual interference interval as $\varphi =0.5{}^{\circ }$, and the null broadening as $4{}^{\circ }$. It can be seen from Simulation 1 that with the decrease in *k*, the null of the beamforming diagram gradually deepens, and the output SINR gradually decreases. It is because the reduction in power parameter *k* causes the increase in the proportion of interference in SINR, resulting in a stronger degree of interference suppression. However, the reduction in noise power leads to the reduction in noise suppression. According to the MVDR criterion, the output SINR decreases slightly.

**Simulation** **2.** *Different φ performances.*

**Figure 5:**
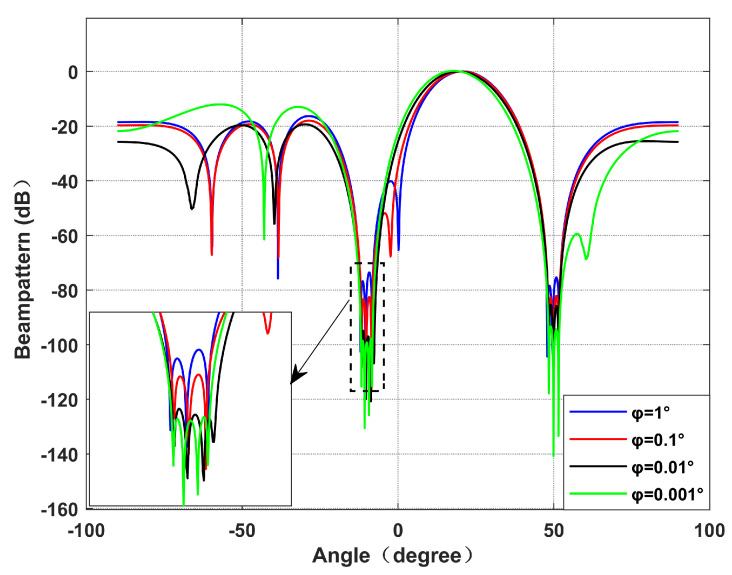
Beamforming diagram with different values of φ. Simulation 2.

**Figure 6:**
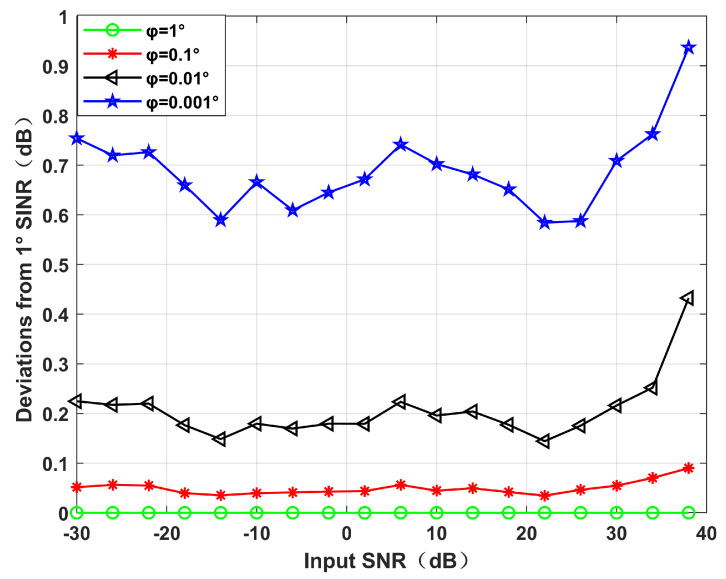
Decrease between each SINR and $1^{\circ }$ virtual interference interval. Simulation 2.

Simulation 2 simulates the beamforming diagram, and the output SINR changes with the input SNR during different φ. We set the power parameter as k=0.2, and the null broadening as $4{}^{\circ }$. It can be seen from Figure 5 that with the decrease in φ, the null becomes deeper and deeper. This is because increasing virtual interference is equivalent to increasing the proportion of interference. Figure 6 shows the decrease in the output SINR between each virtual interference interval and $1^{\circ }$ virtual interference interval. It can be seen intuitively from Figure 6 that with the narrowing of the virtual interference interval, the output SINR gradually decreases.

**Simulation** **3.** *Different null broadening range performances.*

**Figure 7:**
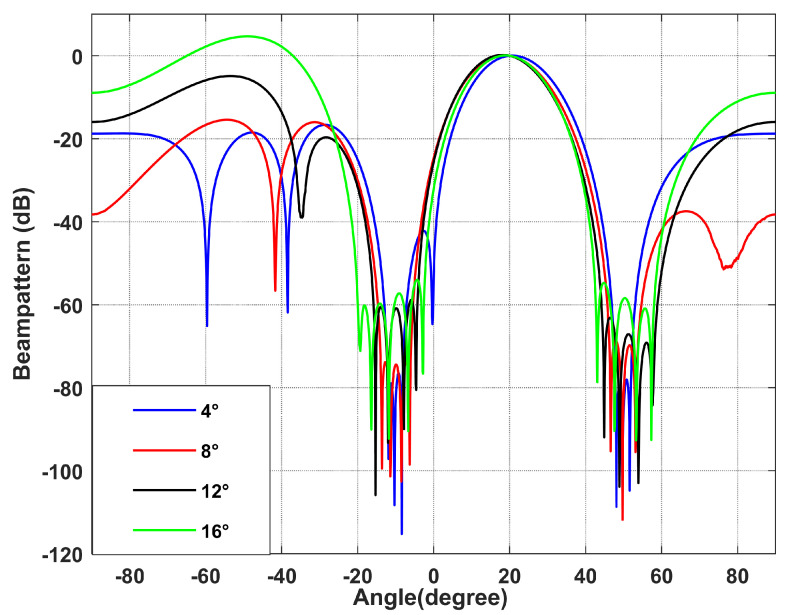
Beamforming diagram with different null broadening angles. Simulation 3.

**Figure 8:**
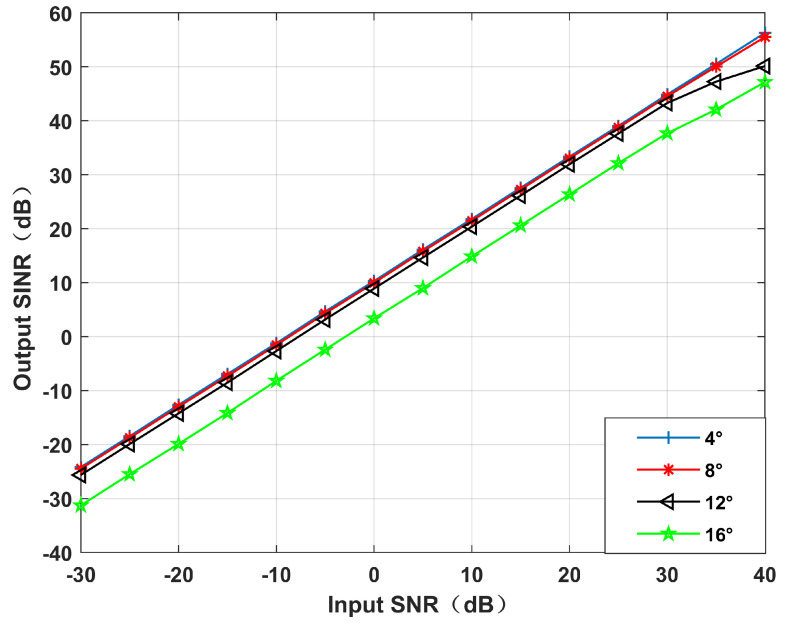
Output SINR versus the input SNR for different null broadening angles. Simulation 3.

Simulation 3 simulates the beamforming diagram, and it can be seen that the output SINR changes with the input SNR during different null broadening ranges. It can be seen from Figure 7 and Figure 8 that when the null broadening range is $4^{\circ }$, the null depth is the deepest, the side lobe is the lowest, and the output SINR is the highest. With the widening of the broadening range, the null depth becomes shallow, the level of the side lobe increases gradually, and the output SINR decreases gradually. In particular, when the null broadening range is $16{}^{\circ }$, the side lobe exceeds the main lobe, and the algorithm performance drops significantly. The output SINR reduces by 10 dB more than $4^{\circ }$. Therefore, if the prior information in the direction of interference movement can be obtained, we can achieve better performance by setting a narrower null width with the same interference suppression effect.

In order to determine the working conditions of the algorithm in this paper under different conditions, we simulated the working conditions under different power and signal angles, and summarize them in Table 3.

### 4.2. Comparison of the Proposed Algorithm and Other Algorithms

In this section, the proposed algorithm will be compared against the sampling matrix inversion (SMI) algorithm [4], CMT algorithm [12], PDL algorithm [17], interference add noise cancellation difference matrix reconstruction (CMRSC) algorithm [19], and the literature [20] algorithm. The parameter settings in each comparison algorithm are consistent with those of the references. At the same time, optimal SINR is added when comparing SINR. We set the power parameter as k=0.2, the null broadening as $4{}^{\circ }$, the interference range as $\left[-12{}^{\circ },-8{}^{\circ }\right]$ and $\left[48{}^{\circ },52{}^{\circ }\right]$, and the virtual interference interval as $\varphi =0.5{}^{\circ }$. There are four intervals of $\varphi =0.5{}^{\circ }$ on both sides of each actual interference source.

**Simulation** **4.** *Effect of beamforming diagram.*

**Figure 9:**
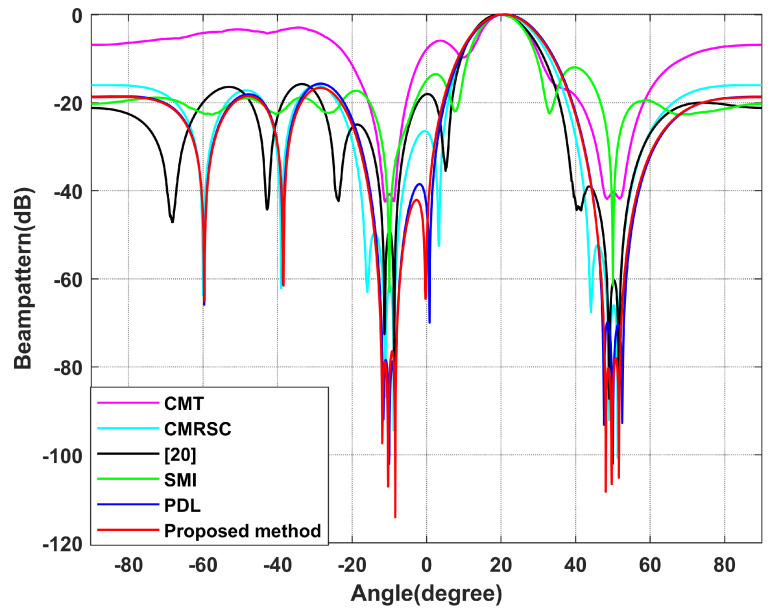
Beamforming diagram. Simulation 4.

**Figure 10:**
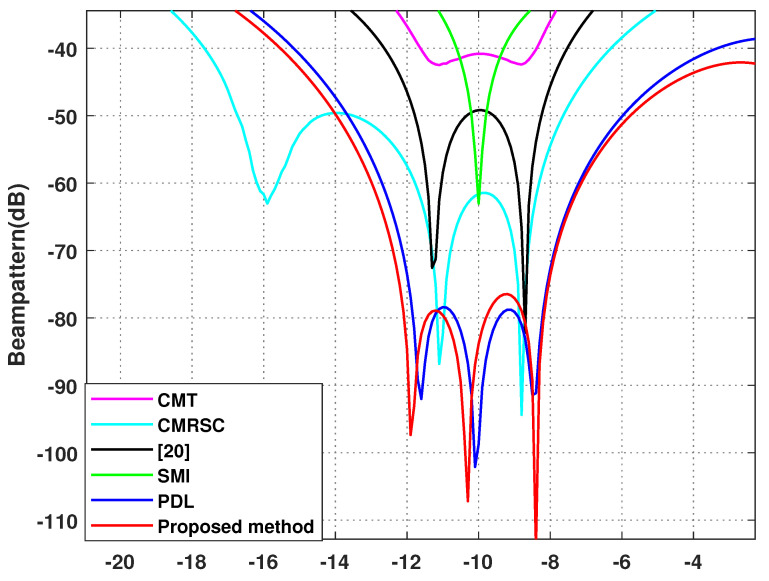
Local enlargement of $-10{}^{\circ }$. Simulation 4.

**Figure 11:**
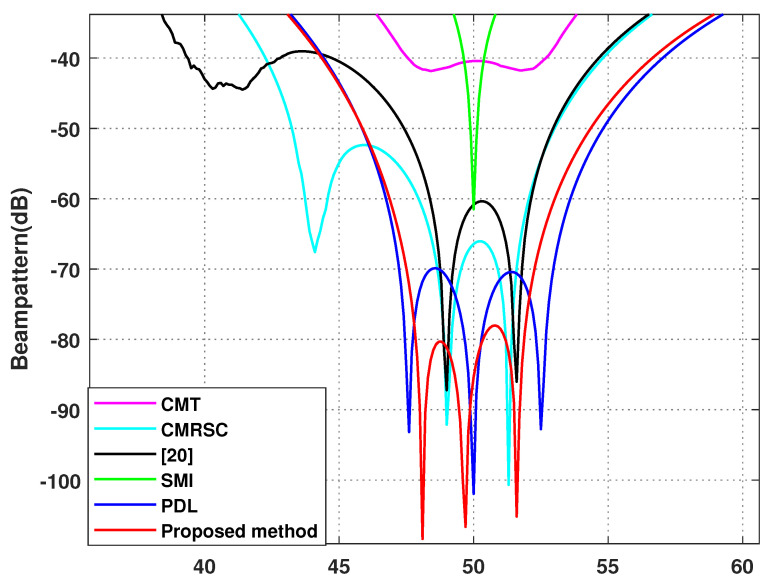
Local enlargement of $50{}^{\circ }$. Simulation 4.

Simulation 4 contrasts the beamforming diagram of each algorithm. Figure 9 is the overall beamforming diagram. Figure 10 and Figure 11 are local beamforming diagrams with $-10{}^{\circ }$ and $50{}^{\circ }$. We can intuitively see that the null depth of the CMT algorithm is the shallowest and the side lobe is the highest. The null depth of the proposed algorithm at $-10{}^{\circ }$ is equal to the PDL algorithm. The deepest null depth is $50{}^{\circ }$. The algorithm proposed in this article is stronger than the interference, in the exhibition area, and at least has a null depth of −77 dB. At the same time, the side lobe of this algorithm is the lowest.

**Simulation** **5.** *Output SINR performance.*

**Figure 12:**
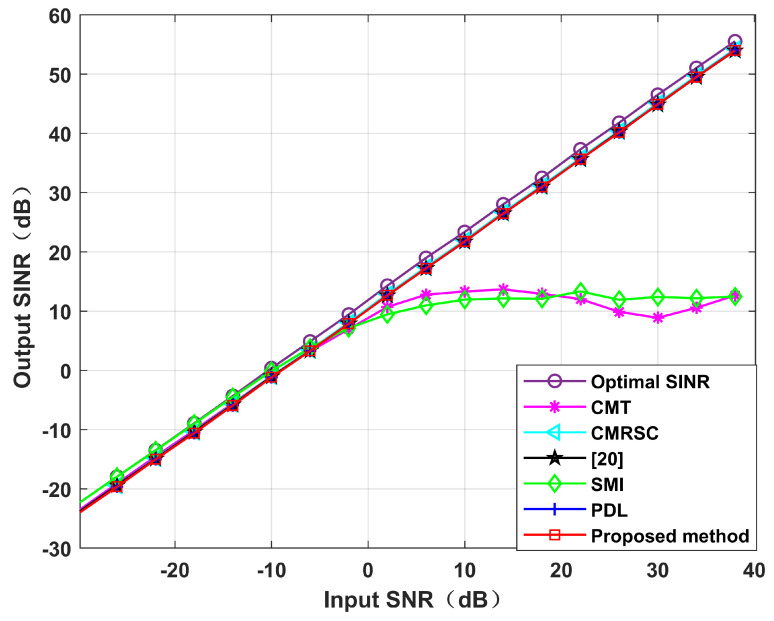
Output SINR under different input SNR conditions. Simulation 5.

**Figure 13:**
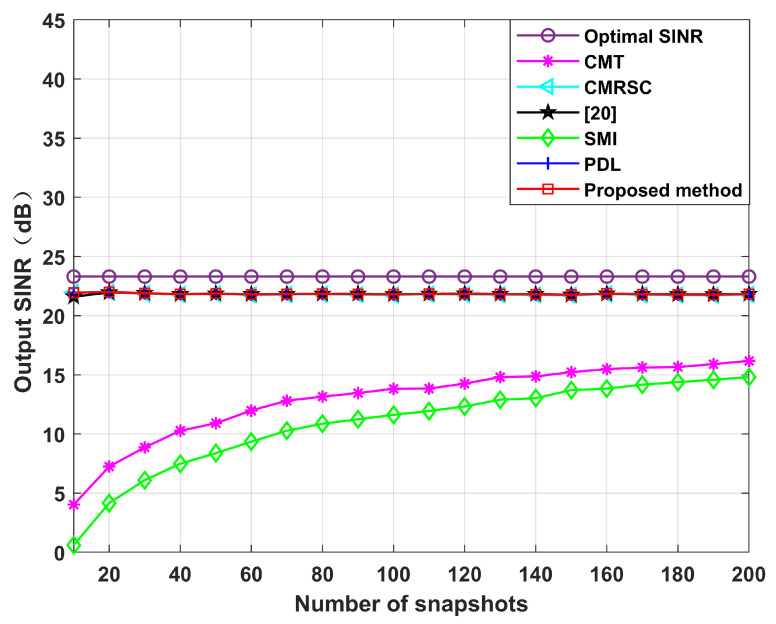
Output SINR under different numbers of snapshots. Simulation 5.

Simulation 5 simulates the impact of the input SNR and the number of snapshots. Figure 12 sets the input SNR as [−30,40], and the number of snapshots as 100. It can be seen from Figure 11 that the performance of each null broadening algorithm is similar in the case of low SNR. The CMT algorithm is the worst in the case of high SNR. The proposed algorithm is comparable to other algorithms, being close to the theoretical optimal value. Therefore, the decrease in complexity of this paper is not exchanged at the expense of sacrificial performance. Figure 13 sets the number of snapshots as [10,200], and the input SNR is 10 dB. It can be seen from Figure 13 that the CMT algorithm is the worst, and the proposed algorithm is comparable to other algorithms. Thus, the number of snapshots has little influence on the algorithm in this paper.

**Simulation** **6.** *Impact of the SV mismatch on the algorithm.*

All of the above simulations have the same signal direction vector as a priori information, ensuring accuracy in the DS direction vector. However, when the desired signal SV is unknown or the desired signal direction is offset due to array disturbance, the robustness of the algorithm to the SV mismatch of the desired signal becomes extremely important.

**Figure 14:**
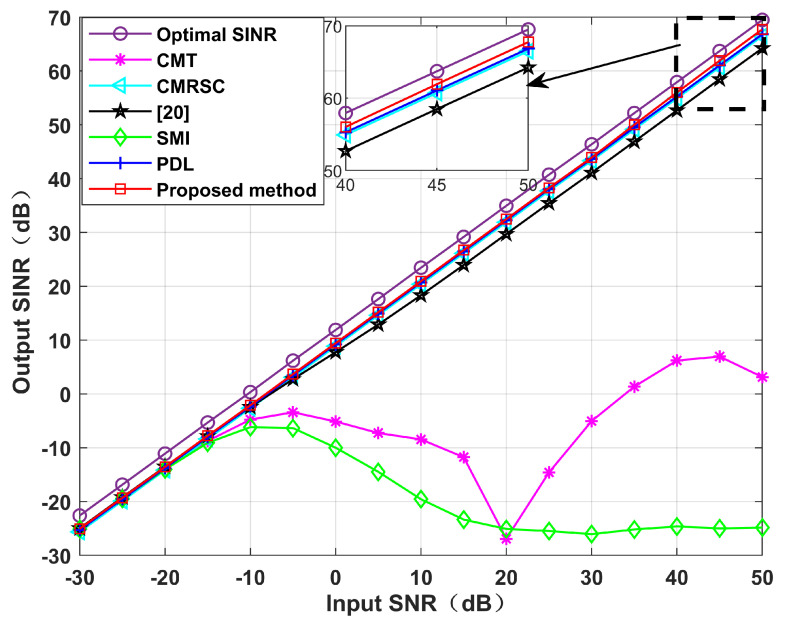
Output SINR when there is a $+5{}^{\circ }$ desired signal SV mismatch. Simulation 6.

**Figure 15:**
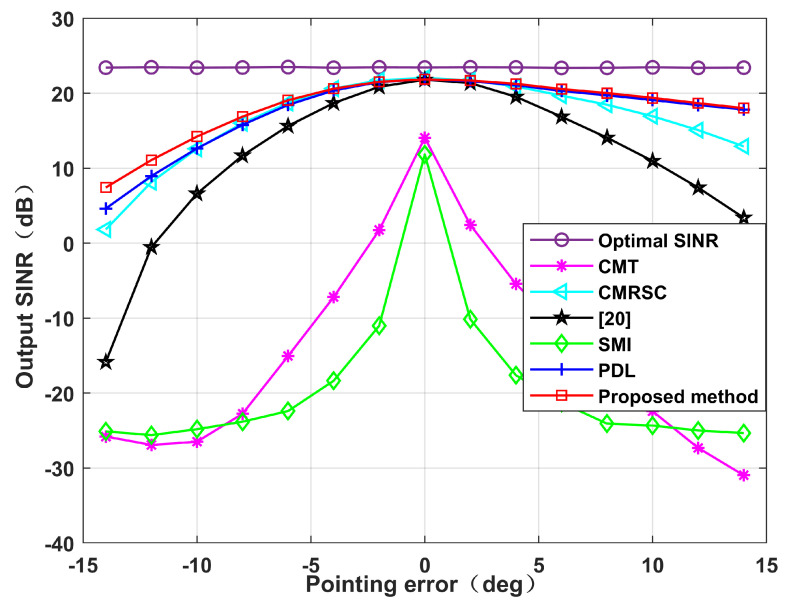
Output SINR at different mismatch angles. Simulation 6.

**Figure 16:**
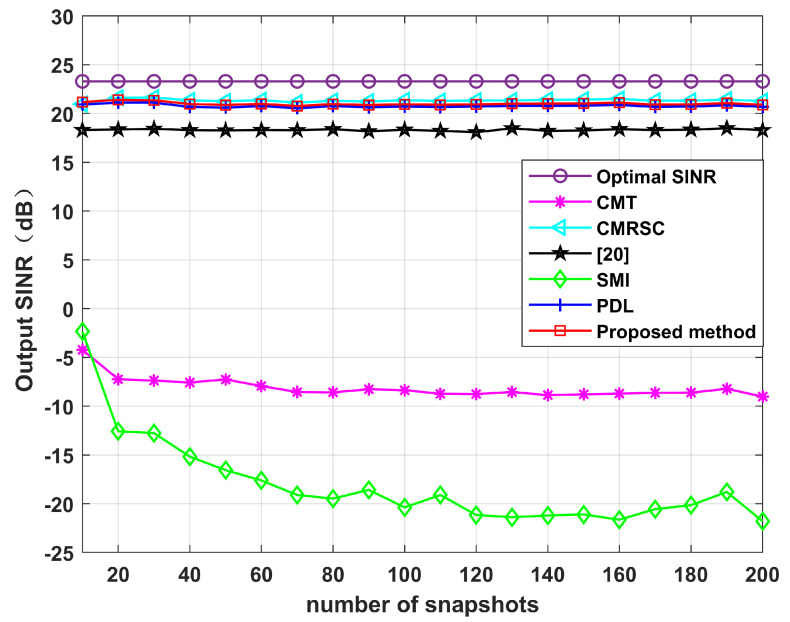
Output SINR versus the number of snapshots in the case of a DS with a $+5{}^{\circ }$ deviation. Simulation 6.

Figure 14 simulates the output SINR changes with the input SNR when the DS has $+5{}^{\circ }$ deviation. We set the input SNR as [−30,50]. It can be seen that in the case of high SNR, the algorithm proposed in this paper is superior to other algorithms. Without loss of generality, Figure 15 simulates the output SINR at multiple mismatch angles. The simulation shows that the proposed algorithm has better performance and stronger robustness for large angles of SV mismatch. Figure 16 displays the output SINR versus the number of snapshots in the case of a DS with $+5{}^{\circ }$ deviation. Setting the number of snapshots as [10,200], and the input SNR as 10 dB, it can be seen that the performance of the proposed algorithm is equivalent to that of the CMRSC algorithm and PDL algorithm. Moreover, it is superior to other algorithms.

**Simulation** **7.** *Impact of array geometry errors.*

**Figure 17:**
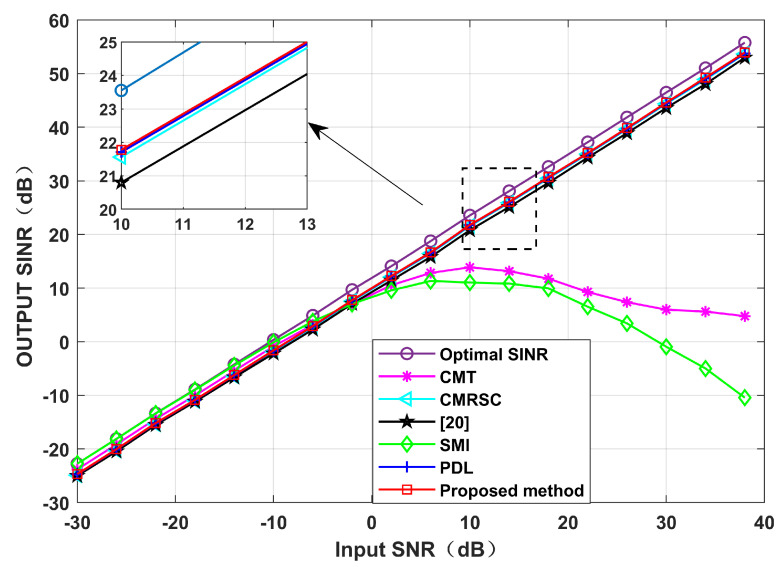
Output SINR versus input SNR in the case of array geometry errors. Simulation 7.

**Figure 18:**
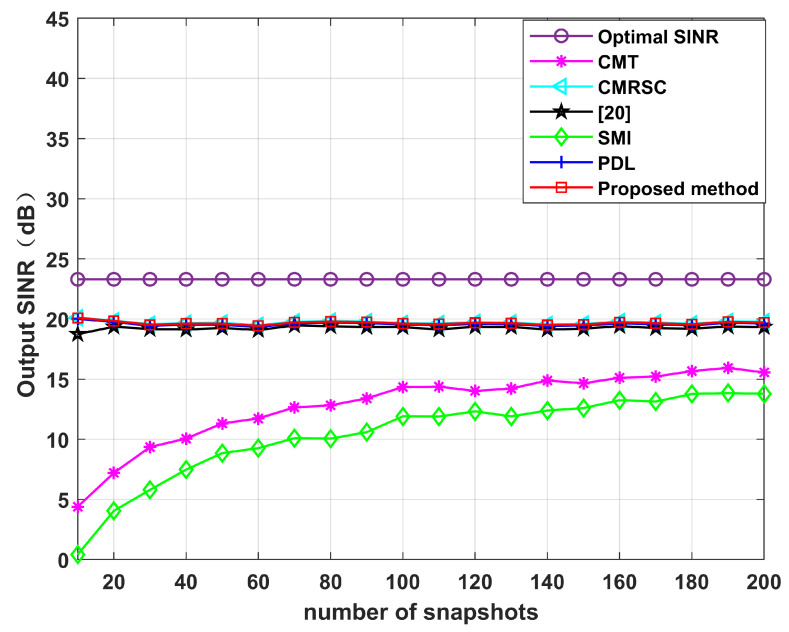
Output SINR versus the number of snapshots in the case of array geometry errors. Simulation 7.

Simulation 7 simulates a case when the array is not perfectly calibrated due to process-level or external forces. In the simulations, the mismatches were modeled as a Gaussian process with a mean of 0 and a variance of 0.01. It can be seen from Figure 17 and Figure 18 that the performance of the algorithm generally degrades when there is an array position error. However, the performance of the proposed algorithm is comparable to that of the mainstream algorithms.

## 5. Conclusions

In this paper, a null broadening algorithm based on power estimation is proposed. First, the proposed algorithm estimates the power of interference according to subspace theory. Then, the correspondence between signal power and direction vector is obtained by calculating the orthogonality of the signal direction vector and eigenvector, and the ICM is obtained according to the definition of the covariance matrix. Finally, the idea of virtual interference sources is introduced to broaden the null. As a result, the algorithm achieves a better performance under the condition of low computational complexity. Simulation results show that, compared with other algorithms, the proposed algorithm has a deeper null, lower side lobes, and stronger robustness under the SV mismatch.

## Figures and Tables

**Table 1:** The proposed algorithm steps.

**Step 1**	Obtain the arrival angle of interference signals $\theta_{2},\cdots ,\theta_{K}$ through Formula (14);
**Step 2**	Find the approximate power $\sigma_{i}^{2}$ of the signal through Formula (23), and the estimated noise power $\hat{\sigma_{n}^{2}}$ is obtained by the Formula (27);
**Step 3**	Set the appropriate exhibition area $\hat{\theta_{i}}$ according to the actual needs;
**Step 4**	Reconstruct the interference-plus-noise covariance matrix ${\hat{R}}_{i+n}$ according to Formula (28);
**Step 5**	Bring ${\hat{R}}_{i+n}$ and $a\left(\theta_{1}\right)$ into the Formula (29), and obtain the weight vector w of this algorithm.

**Table 2:** Comparison of computational complexity.

Algorithms	Computational Complexity
The SMI algorithm [4]	$O(\max\left(M^{2},\left(M^{2}+2M+1\right)M\right))$
The CMT algorithm [12]	$O(\max\left(M^{3},M^{2},\left(M^{2}+2M+1\right)M\right))$
The PDL algorithm [17]	$O(\max\left(M^{3},6M^{2},\left(M^{2}+2M+1\right)M\right))$
The CMRSC algorithm [19]	$O(\max\left(2M^{3},PM^{2},\left(M^{2}+2M+1\right)M\right)),P\gg J,P\gg M$
The literature algorithm [20]	$O(\max\left(M^{3},PM^{2},\left(M^{2}+2M+1\right)M\right)),P\gg J,P\gg M$
The proposed algorithm	$O(\max\left(M^{3},JM^{2},\left(M^{2}+2M+1\right)M\right))$

**Table 3:** Beamforming analysis with different beam pattern gains.

SNR	INR	Direction of Signal Signal (degree)	Direction of Interference Signal (degree)	Null Depth (dB)	Beam Width	Output SINR (dB)	Max SLL
10	30	−20	0, 40	−90.9, −113	16.6	21.36	−10.67
10	30	−40	−10, 30	−112.9, −110.7	20.1	22.26	−13.22
10	30	20	−10, 50	−116.5, −109.5	14.5	22.31	−16.67
10	20	20	−10, 50	−92.53, −68.49	14	21.88	−15.83
0	20	20	−10, 50	−94.44, −70	14.1	10.42	−15.97

## Data Availability

The data that support the findings of this study are available upon request from the authors.
